# Virtual reality cross-sections matched to endoscopic ultrasound for preprocedural simulation of hepaticogastrostomy

**DOI:** 10.1055/a-2740-1053

**Published:** 2025-12-03

**Authors:** Daisuke Namima, Hideki Kamada, Noriko Nishiyama, Naoki Fujita, Hiroki Yamana, Kiyoyuki Kobayashi, Hideki Kobara

**Affiliations:** 1 12850 Department of Gastroenterology and Neurology, Faculty of Medicine, Kagawa University, Kagawa, Japan

Virtual reality (VR) is increasingly being used in clinical practice for preprocedural planning and education 1 2 3 4 . For endoscopic ultrasound (EUS)-guided hepaticogastrostomy, a precise three-dimensional understanding of biliary anatomy is essential 5 . We applied computed tomography (CT)-derived VR planning to enhance preparation for EUS-guided hepaticogastrostomy. Contrast-enhanced CT DICOM data were imported into HoloeyesMD (Holoeyes Inc.) and reviewed on a head-mounted display (Meta Quest 3; Meta Platforms, Inc., Menlo Park, CA, USA) ( Fig. 1 ). The VR environment allowed extensive manipulation: organ filters enabled selective visualization of target structures ( Fig. 2 ), and arbitrary cross-sectional planes could be created and verified on the fly to align the model with the anticipated endosonographic view.

**Fig. 1:**
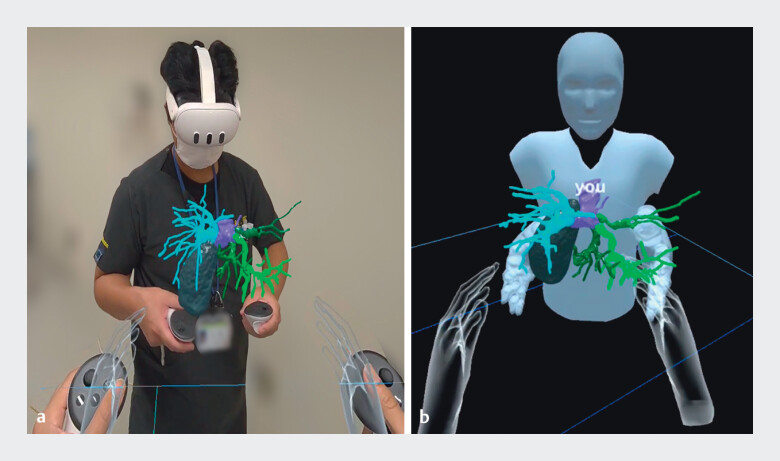
Virtual reality visualization. A clinician uses a head-mounted display to review and manipulate a three-dimensional anatomical model. **a** A clinician wearing a Meta Quest 3 operates the model. **b** A remote collaborator simultaneously views and manipulates the same virtual reality scene within a shared virtual space.

**Fig. 2:**
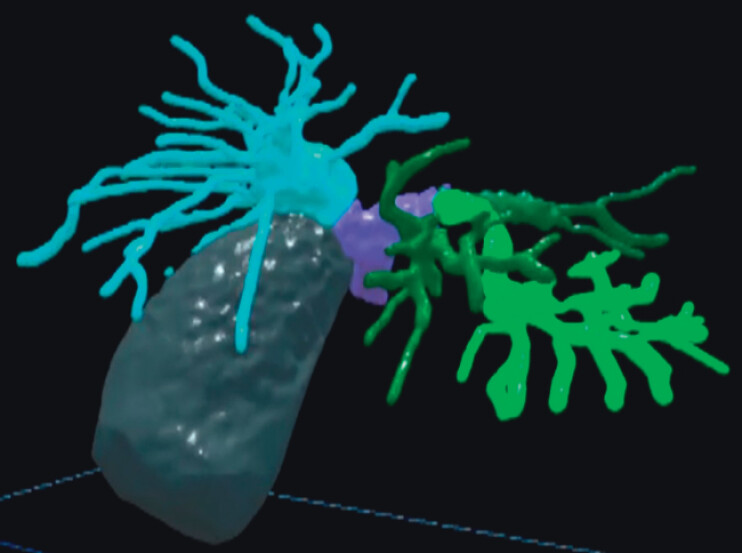
Computed tomography-derived virtual reality biliary model. Organ filters enable selective visualization of specific organs or regions, and color-coding facilitates intuitive understanding of anatomical relationships.

This approach was used in a 76-year-old patient with pancreatic head adenocarcinoma after failed endoscopic retrograde cholangiopancreatography. In VR, we reproduced the expected EUS plane, identified B3 as the target duct, and simulated the needle trajectory ( Fig. 3 ). Switching to a whole-tree view clarified the duct caliber, curvature, distance to the common bile duct, and relationships with adjacent organs, helping predict guidewire direction and avoid unfavorable angles. Quantitatively, the gastric wall-to-B3 distance in VR (3.07 cm) closely matched the intraprocedural EUS on-screen caliper value (approximately 3.0 cm) ( Fig. 4 ).

**Fig. 3:**
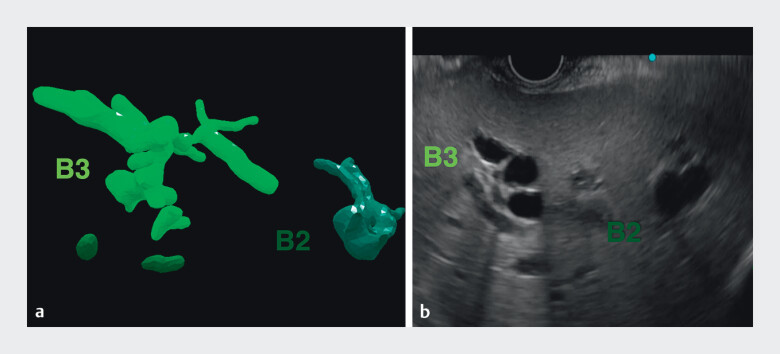
Matched cross-sections. Arbitrary cross-sectional planes generated from the virtual reality model enable simulation of the puncture line. **a** virtual reality-derived biliary cross-section reproducing the anticipated endoscopic ultrasound plane. **b** Corresponding intraprocedural endoscopic ultrasound image.

**Fig. 4:**
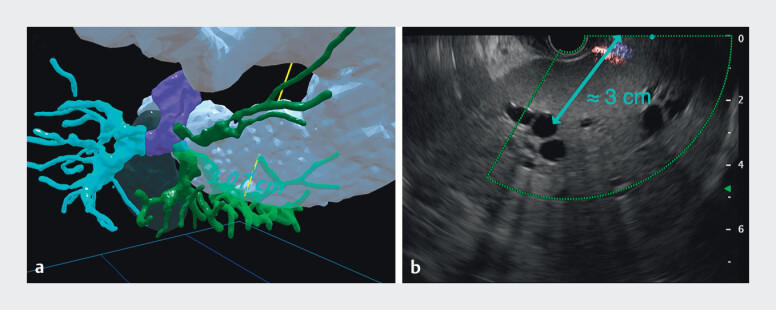
Quantitative validation of the virtual reality plan (gastric wall-to-B3 distance). **a** Virtual reality caliper measurement along the planned trajectory (3.07 cm). **b** Intra-procedural endoscopic ultrasound on-screen calipers measuring the same distance (approximately 3.0 cm), showing close agreement.

Under linear EUS and fluoroscopy, B3 was punctured; cholangiography, smooth guidewire advancement into the common bile duct, tract dilation, and plastic stent placement were all completed successfully with no intra- or postprocedural adverse events. A post-placement VR model showed visual concordance between the planned trajectory and the final stent path ( Video 1 ). CT-derived, headset-based VR planning may enhance spatial cognition, support safer trajectory selection, and improve efficiency while also offering practical benefits for team communication, training, and remote collaboration.

Preprocedural virtual-reality simulation was used to plan endoscopic ultrasound–guided hepaticogastrostomy, which was subsequently performed.Video 1

Endoscopy_UCTN_Code_TTT_1AS_2AH

## Notes

Endoscopy E-Videos https://eref.thieme.de/e-videos
*E-Videos* is an open access online section of the journal *Endoscopy* , reporting on interesting cases and new techniques in gastroenterological endoscopy. All papers include a high-quality video and are published with a Creative Commons CC-BY license. Endoscopy E-Videos qualify for HINARI discounts and waivers and eligibility is automatically checked during the submission process. We grant 100% waivers to articles whose corresponding authors are based in Group A countries and 50% waivers to those who are based in Group B countries as classified by Research4Life (see: https://www.research4life.org/access/eligibility/ ). This section has its own submission website at https://mc.manuscriptcentral.com/e-videos .
